# Potential impact of restricted caribou (*Rangifer tarandus*) consumption on anemia prevalence among Inuit adults in northern Canada

**DOI:** 10.1186/s40795-019-0292-9

**Published:** 2019-05-16

**Authors:** Tiff-Annie Kenny, Xue Feng Hu, Jennifer A. Jamieson, Harriet V. Kuhnlein, Sonia D. Wesche, Hing Man Chan

**Affiliations:** 1 Department of Biology, University of Ottawa, 30 Marie Curie Private, Ottawa, ON K1N 6N5 Canada; 2 Department of Human Nutrition, St. Francis Xavier University, 2320 Notre Dame Avenue, Antigonish, NS B2G 2W5 Canada; 3 Centre for Indigenous Peoples’ Nutrition and Environment, McGill University, 21111 Lakeshore Road, Ste-Anne-de-Bellevue, H9X 3V9 QC Canada; 4 School of Dietetics and Human Nutrition, McGill University, 21111 Lakeshore Road, Ste-Anne-de-Bellevue, H9X 3V9 QC Canada; 5 Department of Geography, Environment and Geomatics, University of Ottawa, 60 University, Ottawa, ON K1N 6N5 Canada

**Keywords:** Inuit, Indigenous, Anemia, Hemoglobin, Animal source food, Traditional food, Country food, Nutrition security, Food security

## Abstract

**Background:**

Caribou (*Rangifer tarandus*) is the top dietary source of iron and several micronutrients necessary for red blood cell production (erythropoiesis) in the contemporary diet of Inuit adults across Canada. Many caribou populations across the circumpolar north, however, have experienced dramatic declines in recent decades. Restricted access to caribou may negatively impact the nutrition and health of Inuit communities.

**Methods:**

We used data from the Inuit Health Survey, a cross-sectional survey of 2550 Inuit adults in thirty-six communities across northern Canada (conducted in 2007–2008) to examine the relationship between caribou consumption, hemoglobin (Hb), and blood biomarkers of nutrient intake and contaminant exposure. Multivariable linear regression was used to investigate the potential public health impact of a theoretical restriction in caribou consumption, by estimating the response of Hb concentrations (and the attendant change in anemia prevalence), to theoretical changes in caribou consumption (with and without substitution of caribou with other country food meat).

**Results:**

Mean (95% CI) daily caribou meat consumption differed by an order of magnitude 4.3 (3.9–4.7), 51.1 (48.5-53.8), and 236.7 (224.7–248.7) grams/day between tertiles of caribou consumption. Mean (95% CI) hemoglobin levels increased from 129.1 (128.1–130.2) g/L to 132.5 (131.3–133.7) g/L between the highest and lowest tertiles of caribou consumption. In multivariable regression analyses, average daily caribou meat consumption was positively associated (*P*< 0.001) with hemoglobin levels. This relationship translated into approximately 4 g/L hemoglobin increase in participants in the third tertile of caribou consumption. The overall prevalence of anemia observed in the study population was 26.5% (24.5 – 28.3%) and a modelled restriction in caribou consumption (i.e. caribou = 0) increased the overall prevalence of anemia by approximately 6%. The maximum negative effect of caribou restrictions was related to a complete restriction on caribou consumption, coupled with the substitution of caribou with other country food meat (35.4% prevalence).

**Conclusions:**

Given the importance of caribou to Inuit culture, health and wellbeing, and the high price of healthful market foods in remote northern communities, strategies to promote the sustainable harvest of country foods are urgently required to ensure the health and nutrition security of the Inuit, in the context of rapidly changing Arctic environments and ecosystems.

## Background

Anemia is a major international health issue affecting an estimated 1.62 billion people globally (95% CI: 1.50–1.74 billion), almost a quarter (24.8%) of the world’s total population [1]. Moreover, anemia is disproportionately represented in people of lower socioeconomic strata [2], and Indigenous Peoples in various global regions [3]. Among Inuit (Arctic Indigenous People) in northern Canada, the prevalence of anemia is several times higher than the average prevalence documented in many developed countries (approximately 8%) [4], and corresponds to a moderate (25–30% prevalence) to severe (40–43% prevalence) public health problem [5–7].

Approximately half of all cases of anemia on a global scale are assumed to be caused by dietary iron deficiency, related to inadequate iron intake, poor iron bioavailability, high iron needs, or high loss of iron [8]. However, the etiology of anemia is multifactorial and context-specific (e.g. population, region, and general environmental), with nutrient deficiencies, malaria, infections, inflammatory disorders, blood disorders, and low socioeconomic status, counted among its most frequent causes [8].

The declining consumption of country foods (i.e. wild foods harvested by Inuit using cultural knowledge, and traditional practices) may place Inuit at increased risk for both iron deficiency and anemia [9]. Country foods obtained from hunting, fishing, and trapping, remain fundamental to the Inuit food system, despite rapid sociocultural and economic changes in Inuit communities over the last several decades [10]. While Inuit harvest and consume a diversity of local species (including both plant, and animal species) for culture and subsistence, dietary studies show that caribou (*Rangifer tarandus*) generally represents the most frequently, and abundantly, consumed country food [11, 12]. Over 90 % of participants in the Inuit Health Survey reported consuming caribou over the previous year, with a mean annual consumption of 29.6–122.8 kg of caribou per person, according to sex, and region [12]. Caribou is also the number one source of iron (up to 36.5% of total population iron intake) and several micronutrients (including zinc, copper, vitamin B_6_, and vitamin B_12_) involved in erythropoiesis reported in contemporary Inuit diets [11, 13].

Many caribou populations across the circumpolar north, however, have experienced dramatic declines (70–97%) in recent decades [14–17], prompting restrictions on Inuit subsistence harvest in several regions of the Canadian north [11]. While Inuit have long adapted to fluctuating cycles of species abundance and migration patterns, the declining use of country foods – which may be related to, or exacerbated by, species declines – may place communities at increased risk of food insecurity and public health issues. Barriers to country food harvest and consumption, whether through species decline (i.e. availability), harvest regulations (i.e. accessibility), and/or other sociocultural, economic and environmental factors that restrict availability and access, are of concern to human health, including (but not limited to) food security and the decline of critical nutrients in the diet. Although many nutrients (e.g. protein) may be provisioned from consumption of alternate country food species [18–20] and/or healthful market foods, certain micronutrients may be limitedly available and/or “unaffordable” in the northern food supply [21]. For instance, when caribou is substituted for other country food species (e.g. muskox (*Ovibos moschatus*)), intake of zinc, and in some cases iron and vitamin D, is markedly reduced [19]. Furthermore, the high price of animal-source foods in remote northern community stores, may favour the substitution of caribou with lower-cost high-energy density but low nutrient-density foods such as starches and simple carbohydrates [21].

The human health impacts of wildlife declines and harvest restrictions among Arctic Indigenous Peoples are unknown. The goal of this research is to examine the potential human health impact of restricted caribou consumption for Inuit adults in northern Canada. As caribou is a major source of nutrients important in the prevention of anemia, the specific objectives of this research are to: (i) examine the relationship between caribou consumption, hemoglobin (Hb), and blood biomarkers of nutrient status and contaminant exposure; and (ii) examine the theoretical public health impact of restricted caribou consumption, by modelling the response of Hb to caribou intake using multivariable regression. We hypothesize that caribou consumption is positively associated with blood Hb concentrations; thus, restricting caribou intake will lead to an increased prevalence of anemia in the study population.

## Methods

### Survey design, study setting, and participants

**Fig. 1:**
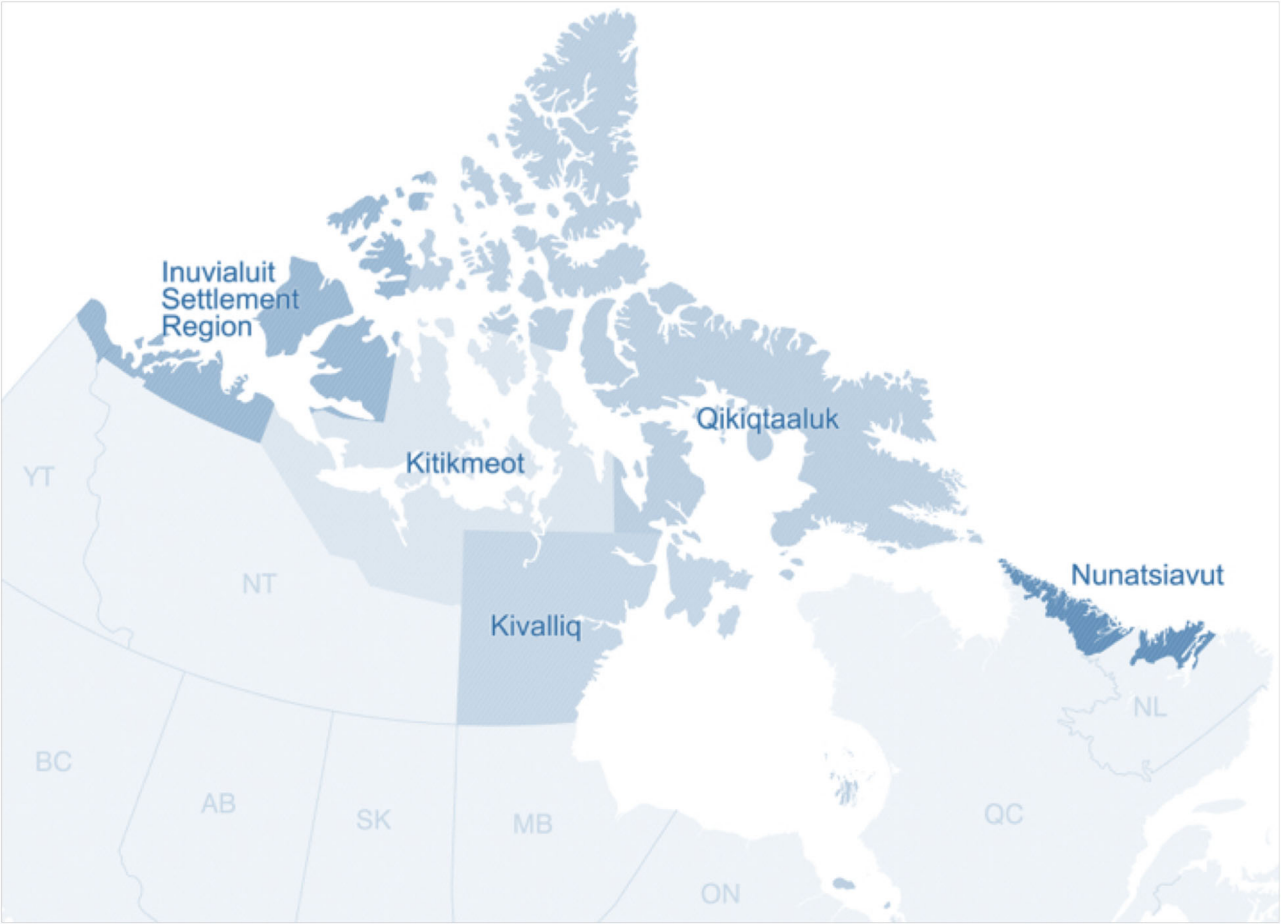
Map of the participating Inuit regions of the 2007–8 Inuit Health Survey. *Nunavut is comprised of the Kitikmeot, Kivalliq and Qikiqtaaluk regions*

Dietary data and blood biomarkers were derived from the 2007–8 International Polar Year (IPY) Inuit Health survey (IHS), a cross sectional health survey of Inuit adults (men and non-pregnant women) residing across three regions of northern Canada (Nunatsiavut, Nunavut, and the ISR; Fig. 1). Detailed methodology for the IHS, including the participatory survey design, has been reported elsewhere [22]. The survey took place between the late summer and fall of 2007 and 2008 in thirty-six communities (latitude of 54°10′N to 76°25′N) spanning the three Inuit regions. Households (*n* = 2796) in each community were selected to participate through a stratified random sampling design and, ultimately, 68% (1901) agreed to participate. All non-pregnant Inuit adults (18 years and older) from the households were eligible to participate in the survey. A total of 2595 self-identified Inuit adults agreed to participate in the IHS, among whom 2169 completed the individual questionnaire and provided blood samples (overall response rate = 83.6%). The sample size was consistent with the 2000 individuals needed to provide statistical power, meet international sample size requirement, and be representative of the total estimated adult population (17,726) in the 3 survey regions at the time of the survey [22]. Informed consent was obtained from all participants. Ethical approval for the IHS was granted by McGill University (Faculty of Medicine Institutional Review Board) and scientific Research Licenses were obtained, where necessary, from northern research institutions (the Aurora Research Institute (Northwest Territories) and (Qaujisaqtulirijikkut (NU)). The University of Ottawa (Health Sciences and Science Research Ethics Board, file number H05–15-16), granted ethics approval for secondary analysis of the data.

### Dietary assessment

Dietary assessments were conducted in-person, by trained interviewers, in English and Inuit Languages. A semi-quantitative food frequency questionnaire (FFQ) was administered to estimate the frequency (number of times per day, week, and month) and usual serving size of each country food item (species, and in some cases, distinct parts such as organs and fat) consumed during the previous year, both in and out of harvest season. The FFQ was developed with input from the IHS steering-committees, based on a revised version of the Centre for Indigenous People’s Nutrition and Environment’s Inuit traditional FFQ. Average (annual average) daily consumption (grams per day, g/d) was calculated for each participant as the product of daily intake frequency and serving size, adjusted for seasonality, according to wildlife harvest calendars. Consumption results were truncated at the 90th percentile to correct for unrealistic reporting. Average daily caribou meat consumption (g/d) was calculated for each participant as the sum of caribou (raw) meat, and caribou dry meat.

Caribou dry meat was converted to fresh weight by correcting for the moisture content differential between raw and dried caribou meat (0.73 and 0.27%, respectively [23]). Total meat from all other country food species, including meat from both terrestrial mammals (e.g. muskox meat) and marine mammals (e.g. ringed seal meat), was summed as “total other country food meat” and included as a model covariate.

### Biochemical assessment

As described in greater detail elsewhere [6, 24, 25], blood biomarkers of nutrient status and contaminant exposure were quantified from fasting (8–16 h preceding the interview) venous blood samples. Blood samples were collected by certified nurses in vacutainer tubes with clot activator and polymer gel for serum separation (Becton Dickinson), or EDTA-coated vacutainers for whole blood hematology. Hb from venous blood samples (morning participants) and blood drops from a finger prick (afternoon participants) was determined using the azide methemoglobin method with a HemoCue 201+ portable photometer (HemoCue; Lake Forest, California). Serum ferritin (SF) and high-sensitivity C-reactive protein (hs-CRP) were quantified by automated chemiluminescence assay (Liaison Ferritin; Diasorin, Italy) and by auto-analyzer (Beckman Coulter; Brea, California), respectively [6]. Soluble transferrin receptor (sTfR) levels were determined by ELISA (R&D Systems; Minneapolis, Minnesota) for a subsample of the population (*n* = 1039). *Helicobacter pylori* (*H. pylori*) seropositivity was ascertained from detection of serum IgG antibodies against *H. pylori* by Immunoenzymatic methods (Calbiotech) at the Montreal General Hospital. Serum 25(OH)D was quantified using the LIAISON total 25(OH)D assay (DiaSorin) at McGill University (detection limit of 0.5 mg/L) as described elsewhere [26]. Fatty acid composition was measured from 200 mL of red blood cells (RBC) stored in 200 mL of a 1:1 solution of methanol and distilled water plus 8.4 mg BHT (Becton Dickinson) based on RBC membranes by Lipid Analytical Laboratories (University of Guelph Research Park). RBC phospholipid FA analyses were expressed as a percentage of total FAs weight. Selenium, mercury, cadmium and lead were quantified in whole blood by an inductively coupled plasma–mass spectrometer (Elan DRC II (Perkin-Elmer SCIEX) for selenium, mercury and cadmium; ELAN 6000 (Perkin-Elmer SCIEX) for Pb) at the Laboratoire de Toxicologie, Institut national de santé publique, (Québec, QC) [25, 27].

### Anemia classification

Anemia was classified according to the World Health Organization (WHO) [2] Hb cutoff values of 130 g/L for men and 120 g/L for non-pregnant women. Cutoff values were adjusted downward (− 0.3 g/L) for current cigarette smokers [2].

### Covariates

Model covariates are summarized in Table 1, including demographic (age, sex, region) and socioeconomic characteristics (e.g. education (post-secondary education), income (> CAD $ 40,000), smoking (current smoker), as well as other factors known or suspected to be involved in the development of anemia among Inuit adults (e.g. consumption of country food meat (non-caribou)), food insecurity and *H. pylori* seropositivity [24]. Food security status was assed using a modified version of the United States Department of Agriculture (USDA) Food Security Survey Module) [28]; food insecurity was classified based on two or more affirmative responses on the 10-item adult scale as recommended by Health Canada [29].

**Table 1:** Demographic, lifestyle, and socioeconomic characteristics according to tertiles of caribou consumption: IPY 2007–2008 Inuit Health Survey

	Tertiles of caribou consumption^1^					
Participant characteristics	Tertile 1		Tertile 2		Tertile 3	
	Mean /n	SD/ %	Mean/n	SD / %	Mean/n	SD / %
Age (year)	43	15	41	14	41	15
Female (%)	491	67	437^*^	60	402^*^	56
Kivalliq (Nunavut)	138	24	137	24	288^*^	51
Qikiqtaaluk (Nunavut)	355	49	235^*^	32	139^*^	19
Kitikmeot (Nunavut)	46	13	118^*^	33	196^*^	54
Inuvialuit Settlement Region	100	37	110	41	57^*^	21
Nunatsiavut	86	34	125^*^	49	45^*^	18
Post-secondary education (%)	138	20	155^*^	22	122	17
Married (%)	429	61	475	66	471	65
Current smoker (%)	500	71	482	67	525	73
Physically inactive (%)	280	39	261	36	202^*^	28
Food insecurity (%)^2^	340	65	282^*^	57	287	62
BMI (kg/m2)	27.6	6	28.6^*^	6	28.9^*^	7
Income > CAD $ 40,000 (%)	140	22	192^*^	30	176^*^	28
*H. pylori* seropositive (%)^3^	481	69	485	68	522^*^	75

*Multiple comparisons between tertiles (tertile 1 vs. tertiles 2 and 3, respectively) with Bonferroni correction. P < 0.05

^1^ Participants were stratified into tertiles based on average (annual average) daily caribou intake g/day, as estimated by the food frequency questionnaire

^2^ Food insecurity includes both moderate and severe food insecurity

^3^ Based on percent inhibition from blood sample

**Table 2:** Average daily consumption of country food^1^, by tertile of caribou consumption (average daily intake - g/day) (*n* = 2175)

	Tertiles of caribou consumption^2^					
	Tertile 1		Tertile 2		Tertile 3	
	Mean	(95% CI)	Mean	(95% CI)	Mean	(95% CI)
Caribou						
Caribou fresh weight^3^	5.6	5.1–6.1	72.7^*^	69.9–75.6	506.8^*^	483.2–530.4
Caribou meat ^4^	4.3	3.9–4.7	51.1^*^	48.5–53.8	236.7^*^	224.7–248.7
Caribou dry meat	0.5	0.4–0.6	8.0	7.2–8.7	99.9^*^	90.8–108.9
Total country food	107.8	97.5–118.0	205.0	194.5–215.5	596.4	576.1–616.7
Other country food meat ^5^	45.9	40.7–51.0	108.3^*^	102.9–113.8	416.0^*^	401.8–430.1
Fish and other seafood ^6^	41.0	35.8–46.1	62.1^*^	56.7–67.5	97.3^*^	90.4–104.2
Fat and muktuk	11.2	8.8–13.6	15.6	13.1–18.0	37.2^*^	32.6–41.7
Plant and berries	3.1	2.6–3.6	4.5^*^	4.0–5.0	6.1^*^	5.5–6.7

*Multiple comparisons between tertiles (tertile 1 vs. tertiles 2 and 3, respectively) with Bonferroni correction, adjusting for age, sex and region of residence. *P* < 0.05

^1^Average daily country food consumption (g/person/day) was based on the food frequency questionnaire and averaged across seasons

^2^ Participants were stratified into tertiles based on average (annual average) daily caribou intake g/day

^3^ Caribou fresh weight calculated based on the sum of caribou meat and caribou dry meat (corrected for moisture content difference)

^4^ Caribou meat - including raw, baked, cooked and aged, preparations

^5^ Aggregated total of meat from all other (non-caribou) country food species, including birds, land mammals (e.g. muskox meat) and marine mammals (e.g. ringed seal meat)

^6^ Does not include marine mammals

### Statistical analysis

Demographic characteristics, country food consumption, and blood biomarkers were described using descriptive statistics (mean (95% CI)). Participants were stratified into tertiles based on average daily caribou consumption (g/day) and multiple comparisons, (with Bonferroni correction) were used to compare means between tertiles. Multivariable linear regression [30] was used to test the hypothesis that caribou consumption was positively associated with blood Hb concentrations. Model covariates (Table 1) were selected a priori based on known, or suspected relationships to Hb in the Inuit population. When necessary, model parameters were logarithmically (ln) transformed to improve normality of the respective distributions. Significance was set at α = 5% for all statistical tests. All statistical analyses were performed with Stata SE® (version 14; StataCorp LP, College Station, Texas).

We investigated the potential human health impact of a theoretical restriction in caribou consumption, by estimating the response of Hb levels to changes in caribou consumption using the multivariable model described above [31]. The population change in anemia prevalence was estimated by subtracting the expected impact of restricted caribou consumptions from baseline Hb concentrations observed in study population. Four scenarios were formulated to evaluate the impact of different levels of restricted caribou consumption and possible adaptation options. Scenario 1 represented a complete restriction on caribou consumption (i.e. caribou intake = 0). Scenario 2 represented a complete restriction on caribou consumption, and substituted caribou meat with the equivalent weight of other country food meat. Finally, scenarios 3 and 4 represented a fifty-percent reduction in caribou consumption, considering both no substitution of caribou meat (scenario 3), and replacing caribou with intake of other country food meat (scenario 4).

## Results

### Participant characteristics

Participant characteristics are presented according to tertiles of caribou consumption in Table 1. Overall, the mean ± SD age of study participants was 42 ± 15 years. The percentage of participants who reported to be current smokers was 70%. The proportion of women decreased from 67 to 56% between tertiles one and three. Likewise, the percentage of physically inactive participants decreased between tertiles one and three (39 to 28%). Participants in higher tertiles of caribou consumption were more likely to report incomes above CAD $40, 000, and *H. pylori* seropositivity. A higher percentage of participants from the Kivalliq and Kitikmeot regions were in the highest tertile of caribou consumption. By contrast, fewer participants from Nunatsiavut, the Qikiqtaaluk region, and the Inuvialuit Settlement Region were represented in the highest tertile of caribou consumption (Table 2).

### Blood biomarkers

Most concentrations of blood nutrients and contaminants remained stable between tertiles (Table 3). Mean (95% CI) concentrations of % eicosapentonoic acid (EPA), magnesium, selenium, and mercury were lower in the third tertile, relative to tertile 1. Mean (95% CI) Hb levels were higher in tertiles two 132.5 (131.3–133.7) g/L, and three 132.9 (131.8–134.1), relative to tertile one 129.1 (128.1-130.2) (Table 4). Similarly, mean (95% CI) serum ferritin concentrations were higher in tertiles two 58.5 (54.1–63.0) g/L, and three 57.5 (53.1–61.9) g/L, relative to tertile one 50.1 (46.3–53.9) g/L (Table 4).

**Table 3:** Blood biomarkers of contaminants and nutrients by tertile of caribou consumption: IPY 2007–2008 Inuit Health Survey (*n* = 2175)

	Tertiles of caribou intake					
	Tertile 1		Tertile 2		Tertile 3	
	Mean	(95% CI)	Mean	(95% CI)	Mean	(95% CI)
Serum vitamin D (nmol/L)	59.4	56.9–62.0	58.6	56.0–61.2	55.6	53.3–57.8
Plasma vitamin B_6_ (ng/mL)	3.5	2.9–4.0	2.9	2.4–3.4	2.8	2.1–3.5
Total n-3 fatty acids (%)	5.8	5.6–6.1	5.7	5.5–5.9	5.6	5.3–5.8
RBC EPA (%)	1.7	1.6–1.9	1.5	1.4–1.6	1.5^*^	1.4–1.6
RBC DHA (%)	2.5	2.4–2.6	2.6	2.5–2.7	2.4	2.3–2.5
RBC magnesium (mg/L)	52.0	50.9–53.1	51.3	50.4–52.2	50.8^*^	49.9–51.7
Blood selenium (μg/L)	331.7	318.3–345.6	306.3	294.3–318.9	314.4^*^	301.7–327.7
Blood mercury (μg/L)	7.4	6.7–8.2	6.3	5.8–6.8	6.9^*^	6.3–7.5
Blood lead (μg/L)	34.7	32.7–36.8	32.8	31.1–34.6	38.1	36.0–40.2
Blood cadmium (μg/L)	1.7	1.6–1.8	1.5	1.4–1.6	1.7	1.5–1.8

*Multiple comparisons between tertiles (tertile 1 vs. tertiles 2 and 3, respectively) with Bonferroni correction, adjusting for age, sex and region of residence. *P* < 0.05

**Table 4:** Blood biomarkers of anemia and iron status by tertile of caribou consumption (*n* = 2175)

	Tertiles of caribou intake^1^					
	Tertile 1		Tertile 2		Tertile 3	
	Mean	(95% CI)	Mean	(95% CI)	Mean	(95% CI)
Hemoglobin (g/L) (*n* = 2175)	129.1	128.1–130.2	132.9^*^	131.8–134.1	132.5^*^	131.3–133.7
Serum ferritin (ng/mL) (*n* = 2095)	50.1	46.3–53.9	58.5^*^	54.1–63.0	57.5^*^	53.1–61.9
Serum soluble transferrin receptor (mg/L) (*n* = 986)	1.5	1.4–1.6	1.4^*^	1.3–1.4	1.4^*^	1.4–1.5
Serum hs-C-reactive protein (mg/L) (*n* = 2086)	3.1	2.7–3.5	2.7	2.3–3.0	2.7	2.4–3.0

*Multiple comparison with Bonferroni correction, adjusting for age, sex and region of residence. *p* < 0.05

### Modelled change in hemoglobin and anemia

In multivariable regression, Hb was positively associated (*P* < 0.001) with caribou consumption after adjustment for covariates (Table 1), with 0.008 g/L Hb increase per daily gram of caribou consumed (Table 5). This translated into approximately 4 g/L Hb increase in participants in the third tertile of caribou consumption (i.e. who reported consuming ~ 500 g/day of caribou, on a wet weight basis). The consumption of other (i.e. non-caribou) country food meat was negatively associated (< 0.001) with Hb (Table 5). Hb decreased by 0.007 g/L per daily gram consumption of other country food meat, which translated into about 0.5 g/L of Hb decrease in participants of the higher tertile of caribou consumption (approximately 80 g).

**Table 5:** Multivariable linear regression^1^ coefficients for hemoglobin, with both dietary and non-dietary determinants as independent variables^2^. Inuit adults: International Polar Year Inuit Health Survey, 2007–2008

	Coefficient	SE	P
Constant	123.741	3.093	0.000
Caribou consumption (g/day)^3^	0.008	0.003	0.009
Other CF consumption (g/day)^4^	−0.008	0.004	0.020
Age (years)	−0.139	0.030	0.000
Male sex	14.243	0.818	0.000
Region	0.869	0.300	0.004
Current smoker	−0.457	0.873	0.601
BMI (kg/m^2^)	0.204	0.061	0.001
Postsecondary education	0.919	0.512	0.073
Married	1.233	0.788	0.118
Income above CAD $ 40,000	−0.011	0.015	0.459
*H. pylori* seropositivity (% inhibition)	−0.263	0.815	0.747
Food insecure	−2.096	0.813	0.010

^1^ Model *R*^2^ = 0.23; Model adjusted *R*^2^ = 0.22

^2^ Sex, region, smoking status, marital status, post-secondary education, income, and food insecurity (includes both moderate and severe food security) were treated as binary or dummy variables

^3^Average daily caribou meat consumption (g/person/day) was based on the food frequency questionnaire and was averaged across seasons. Average caribou meat consumption was expressed on a fresh weight basis as sum of caribou meat and caribou dry meat (corrected for moisture content difference)

^4^ Other country food consumption represented the aggregated total of meat from all other (non-caribou) country food species, including birds, land mammals (e.g. muskox meat) and marine mammals (e.g. ringed seal meat)

**Fig. 2:**
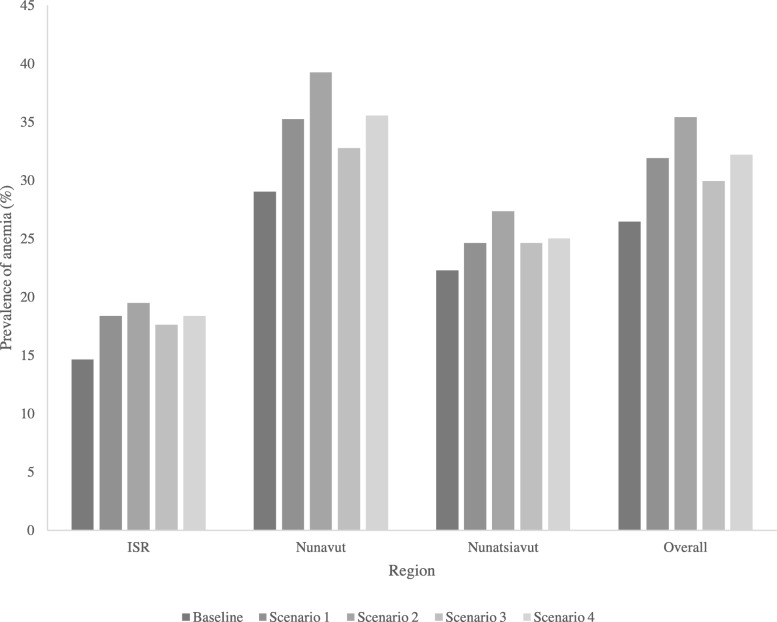
Modelled change in prevalence of anemia among Inuit adults in Canada (by region) from different scenarios of theoretical caribou consumption restrictions. *Description of model scenarios: Baseline: Observed prevalence of anemia in Inuit adults who participated in the 2007–8 Inuit Health Survey, according to WHO cutoff values for hemoglobin, adjusted for smoking. Scenario 1: Complete restriction on caribou consumption (caribou = 0). Scenario 2: Complete restriction on caribou consumption (caribou = 0) and substitution of caribou with other country food meat. Scenario 3: Fifty-percent restriction in caribou consumption. Scenario 4: Fifty-percent restriction in caribou consumption and substitution of caribou with other country food meat*

**Fig. 3:**
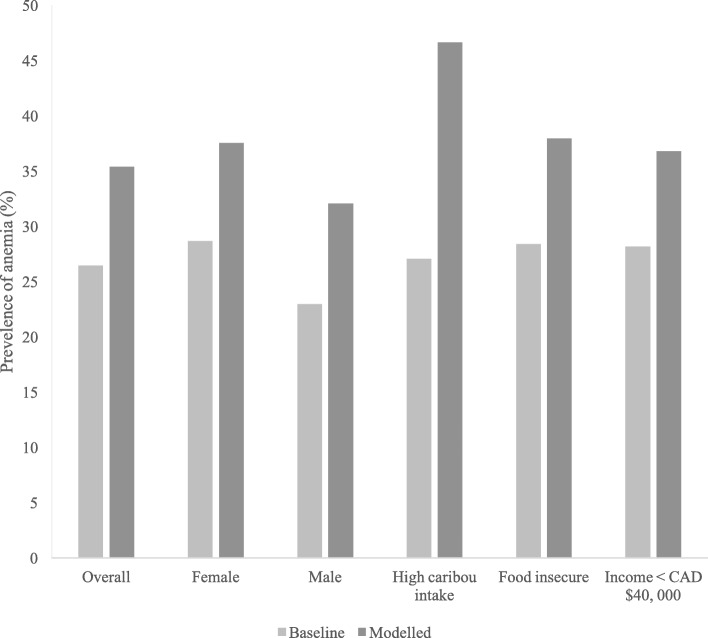
Modelled change in prevalence of anemia according to dietary and socioeconomic factors, from a theoretical restriction of caribou consumption, and the substitution of caribou with other country food meat. *Food insecure combines both moderate and severe food insecurity*

The overall prevalence of anemia in the study population was 26.5% (24.5 – 28.3%) (Fig. 2). The impact of various scenarios of restricted caribou consumption are presented in Fig. 2, based on the observed relationship between Hb and consumption of caribou meat, and other country food meat with (Table 5). A complete restriction on caribou consumption (i.e. restricting caribou to zero in the models) was associated with an overall 31.9% prevalence of anemia in the study population (18.4% in ISR to 35.2% in Nunavut). A complete restriction on caribou consumption, coupled with the substitution of caribou meat with other country food meat, represented the maximum negative effect of caribou restrictions on the population distribution of Hb levels (Fig. 2). This scenario leads to an overall increase in anemia prevalence of approximately 9% (35.4% prevalence) and was higher among females (37.5% prevalence), participants in the highest tertile of caribou consumption (46.6%), and participants with annual incomes below CAD $40,000 (36.9%) (Fig. 3).

## Discussion

Inuit have witnessed climate-related changes on various aspects of their traditional food system**s**, including changes in the accessibility, availability, and condition, of key country food species [18, 32]. In light of emerging livelihood and public health concerns related to caribou declines across northern Canada [33, 34], we have modelled the role of caribou consumption in the maintenance of nutritional adequacy (anemia) for Inuit adults in northern Canada. Previous studies among Inuit adults have reported a positive association between country food consumption, serum ferritin [24], and Hb [6]. The positive association between average daily (annual average) caribou consumption (g/day), serum ferritin, and Hb suggests that barriers to consuming caribou (whether through species decline, associated harvest regulations, and/or other socioeconomic factors that limit subsistence harvests) may represent a concern for public health, including nutrient deficiencies (such as zinc deficiency, associated with diseases such as diabetes and immunological effects [35]) and anemia. While caribou may be substituted for other country food species (e.g. moose or geese), micronutrient levels in the alternate diet may be diminished and result in deficiencies in some individuals [18, 19]. It is important to note, however, that no empirical research has examined changes in nutrition, food security, or public health status, in relation to caribou (or other wildlife) declines and/or related wildlife conservation measures (e.g. harvest quotas) in the Arctic [11]. Given the already high prevalence (up to 68.8%) of food insecurity (i.e. the state of inadequate access to sufficient, safe/nutritious and culturally preferred foods, including country foods) among Inuit in Northern Canada [36], and its adverse association with dietary quality (e.g. higher intake of carbohydrates, and lower intakes of key nutrients such as vitamins C and D, calcium, magnesium, iron, fibre and folate) and blood biomarkers of nutritional status (e.g. lower hemoglobin and serum ferritin levels) documented among Inuit [37], wildlife declines, harvest restrictions, and other factors limiting country food access for Inuit need to be approached (i.e. monitored and responded to, through by appropriate policies and interventions) from a public health perspective.

Although country foods remain vital to the health and wellness of Arctic Indigenous Peoples [10], the public health implications of species declines and harvest restrictions in the Canadian north have not been well studied. While hunting pressures by humans can exacerbate caribou declines, they are not generally recognized as the ultimate cause of population declines [16, 33]. Yet, the health consequences of these declines would be experienced directly by Inuit and other northern Indigenous Peoples who rely on caribou for food security and nutritional adequacy. In Madagascar, wildlife consumption (“bush meat”) contributed approximately 0.7 g/dL to Hb levels in children, and a modelled restriction in bush meat access resulted in an estimated 29% increase in the number of cases of childhood anemia [31]. Nevertheless, even at the international scale, studies on the impact of wildlife depletion and harvest restrictions on food security and human health are limited.

It is important to note that the etiology of anemia among Inuit is not fully understood [5], and recent evidence recognizes non-dietary/nutritional factors (e.g. poverty, household crowding, food insecurity, lead exposure, *H. pylori* infection, inflammation, chronic blood loss, and impaired iron absorption and/or utilization) [6, 38]. There is also concern that exposure to heavy metals (e.g. lead and mercury) from consumption of country foods may expose individuals to unsafe levels of environmental contaminants and represent concern for anemia (e.g. lead interference with heme biosynthesis). Lead exposure among Inuit has been investigated as a potential cause of anemia and has been found to be negatively associated with Hb in men [6]. In this study, most concentrations of blood nutrients and contaminants remained stable between tertiles of caribou consumption. However, we observed that mean concentrations of %EPA, magnesium, selenium, and mercury were lower in the highest tertile of caribou consumption relative to the lowest tertile. It is noteworthy that although country foods are rich sources of heme iron [23], consumption of total other country food meat (i.e. non-caribou country food meat) was negatively associated with Hb in this study. Frequency of marine mammal consumption has been documented as an independent negative predictor of Hb concentrations in the Inuit population of Canada [6]. It is postulated that high red blood cell EPA status, corresponding to higher intake of marine mammals [39], may contribute to anemia in this population [6]. Higher intakes of n-3 fatty acids may alter platelet function and/or hemostasis and lead to increased gastrointestinal blood loss [40]. Thus, high intakes of caribou may reflect lower intakes of marine mammals, contributing to improved iron status and Hb concentrations.

Women of childbearing age, as well as pregnant and lactating women, who are at increased risk of iron deficiency and inadequacies of magnesium and zinc [41, 42] may be at increased risk of adverse health outcomes from restrictions on caribou harvests. This has significant implications for health and social equity, as maternal health and disadvantage have important consequences for intergenerational equity, including child growth and development [43]. In low- and middle-income countries, for example, maternal anemia status is one of the most significant predictors of anemia in children [44]. Although a low prevalence of iron-deficiency anemia has been documented among Inuit children in Nunavut [38], iron depletion was highly prevalent (33%) among Inuit children in Nunavik, where food insecure children also had a greater burden of nutritional deficiencies and slower linear growth [45]. As described above, the human health effects of wildlife depletion have not been well studied, accordingly the public health risks are largely unknown. Public health interventions for the treatment and prevention of iron deficiency anemia generally include iron supplementation and fortification. Nutrient supplementation strategies such as “Sprinkles” [46], for instance, have been included in strategies to prevent anemia in Indigenous children of northern Canada [47, 48]. While anemia among premenopausal Inuit women is largely attributed to low iron /depleted iron stores [6, 7], the vast majority of anemia cases among Inuit men and post-menopausal women are unexplained by iron status [6]. The clinical significance of anemia among Inuit has not, to our knowledge, been established or described in the literature, and requires further study and monitoring – particularly in light of biodiversity declines.

## Limitations

Several important study limitations warrant mention. First, this study is based on cross sectional data which precludes the direct inference of causality. Thus, the relationship between caribou consumption and hemoglobin levels observed in this study warrants further examination through dedicated case studies, that include both epidemiological designs, but also participatory community-engaged methods to capture the many wellbeing and psychosocial impacts of caribou declines on public health. Second, caribou consumption estimated from the FFQ reflects the average diet during the twelve months preceding the interview, while blood biomarkers reflect a single time point (in the late summer/fall). Accordingly, the relationship observed between caribou consumption and blood biomarkers may differ throughout the year. Third, data from the Inuit Health Survey is aggregated at the regional level, which precluded the possibility of linking human health measures to the status of northern caribou populations. Data for all regions was thus combined and modelled at the population level; however, this may have obscured region, age, and sex risk factors [6]. Nevertheless, this is the first study, to our knowledge, that has attempted to establish empirical links between country food consumption and human health status in the Arctic.

## Conclusion

The prevalence of anemia is high in the Inuit adult population of Canada and restricted access to caribou has the potential to further exacerbate this issue. Inuit communities and organizations, conservation scientists, and public health practitioners must work together to implement integrated solutions that ensure the sustainability of caribou populations over the long term, while sustaining food security and public health in the interim. Preventing anemia will also necessitate support to address systemic inequalities (e.g. education, food insecurity and infection) among Inuit [6].

## Abbreviations

BMI: Body mass index
CF: Country food
DHA: Docosahexaenoic acid
EPA: Eicosapentonoic acid
hb: Hemoglobin
hs: High sensitivity
IHS: Inuit Health Survey
RBC: Red blood cell
